# Primary Isoniazid Mono-Resistant Extrapulmonary Tuberculosis Presenting as Cervical Lymphadenitis: The World’s First Case of Its Type

**DOI:** 10.7759/cureus.41937

**Published:** 2023-07-15

**Authors:** Sankalp Yadav

**Affiliations:** 1 Medicine, Shri Madan Lal Khurana Chest Clinic, New Delhi, IND

**Keywords:** cbnaat/xpert/rif assay, mtb (mycobacterium tuberculosis), tuberculosis, mono-resistance, isoniazid resistance

## Abstract

Tuberculosis is commonly seen in endemic countries. Cases of primary drug resistance are rare. There is a paucity of data related to primary drug resistance at extrapulmonary sites. Herein, a case of primary isoniazid mono-resistant extrapulmonary tuberculosis of the multiple right cervical lymph nodes is presented. This patient reported multiple swellings and discharging sinuses. A battery of investigations with an eye for finding drug resistance led to a definite diagnosis. He was initiated on an anti-tubercular regimen per the national guidelines. A detailed literature search revealed that no such case of primary isoniazid mono-resistant extrapulmonary tuberculosis presenting as cervical lymphadenitis has ever been reported.

## Introduction

Tuberculosis is a disease caused due to infection by an acid-fast bacillus, i.e., *Mycobacterium tuberculosis *[1]. This disease has a profound effect on public health [1]. It mainly presents as pulmonary tuberculosis, where the lungs are involved [2]. However, cases of extrapulmonary tuberculosis are also available in the literature where the infection manifests at an extrapulmonary site [2]. Often, this extrapulmonary seeding happens from the lungs via hematogenous or lymphatic spread [3]. On rare occasions, direct infection at the extrapulmonary site results in the disease. The commonest form of extrapulmonary tuberculosis is cervical tuberculous lymphadenitis, classically known as "scrofula," which has a proclivity for children [4].

Drug resistance is a serious threat to tuberculosis elimination programs [5]. The extent of drug resistance is threatening, with 15953 isoniazid mono- or poly-resistant cases reported in the year 2022 from India [6]. Primary drug resistance is an infrequent condition. However, with the availability of advanced diagnostic labs, easy accessibility of health facilities, and reduced time lag from initial presentation to treatment initiation, there has been an increase in the notification of the total number of primary drug resistance cases [6].

A case of a 26-year-old Indian male is presented who came with multiple swellings on the right side of his neck. A diagnosis of primary isoniazid mono-resistant extrapulmonary tuberculosis of the multiple right cervical lymph nodes was made after a diagnostic work-up and clinical assessment.

## Case presentation

In the year 2021, a 26-year-old Indian male, a daily-wage worker by occupation belonging to a low socioeconomic background, presented with multiple swellings on the right side of his neck. These swellings were insidious in onset and increased in size over the last 20 days. For the past 15 days, these have been associated with pain. Some of these were associated with discharging sinuses and purulent discharge. He had no fever, night sweats, cough, or weight loss. There was no history of tuberculosis in him or among his contacts. Furthermore, he had no history of imprisonment, substance abuse, or staying at night shelters or refugee camps.

A general examination revealed a medium-built man with a pulse of 80 per minute, a blood pressure of 120/78 mm of Hg, a temperature of 98.4°F, oxygen saturation of 99% in room air, and a respiratory rate of 16 breaths per minute.

**Figure 1:**
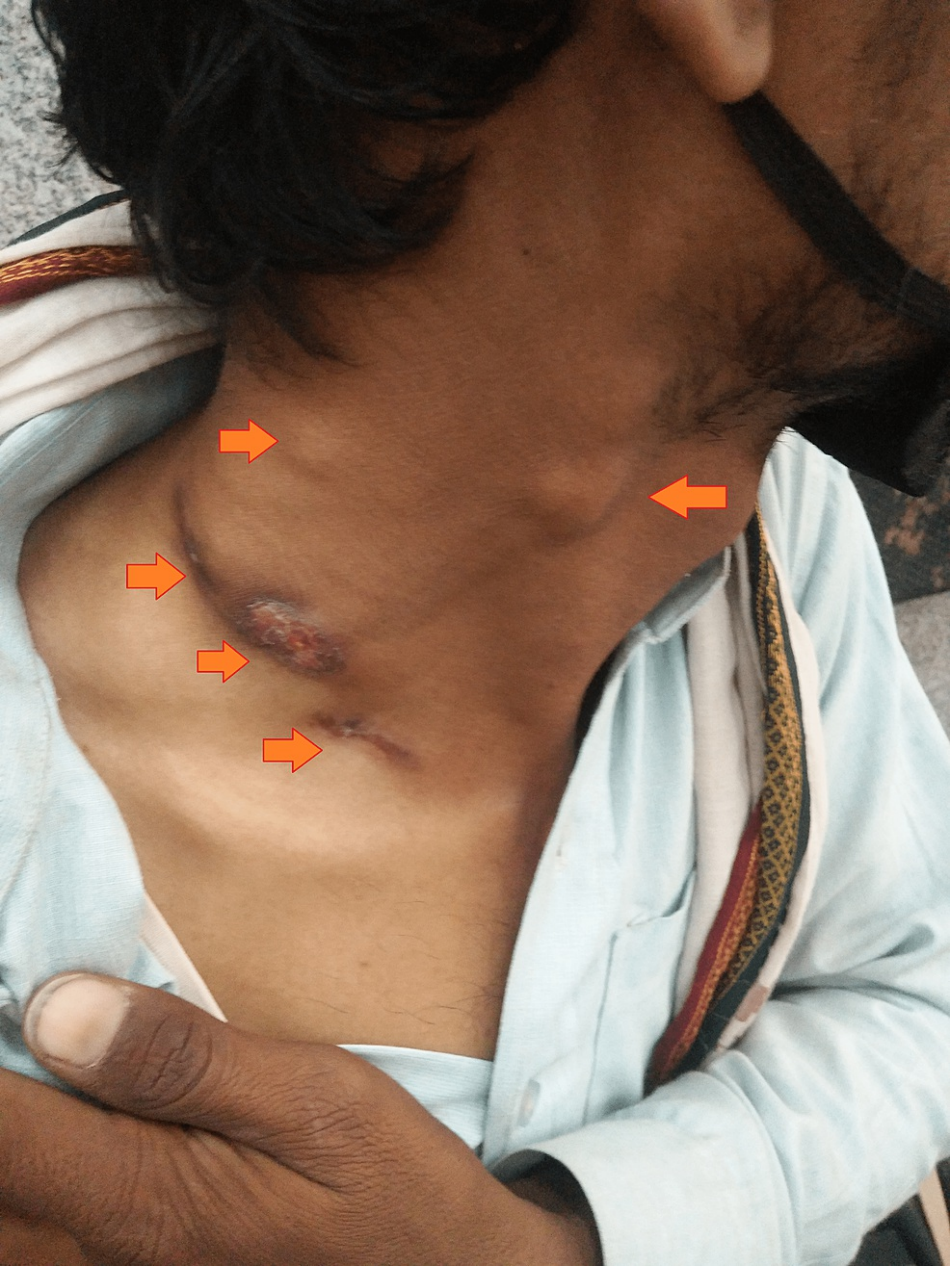
Gross image showing multiple swollen lymph nodes and discharging sinus

The local examination was suggestive of four swollen lymph nodes in the anterior cervical, supraclavicular, and posterior cervical regions. The anterior cervical lymph node was about 2 × 2 cm in size, mobile, firm in consistency, and tender on palpation. The posterior cervical lymph node was about 1 × 1 cm in size, tender on palpation, and firm in consistency. The supraclavicular lymph nodes were ruptured, with a discharging sinus having a yellow-colored, purulent, non-fowl-smelling discharge (Figure 1).

No other palpable nodes were found in the submandibular, axillary, or submental stations. There was no pallor, icterus, clubbing, cyanosis, or edema. A systemic examination was unremarkable.

**Figure 2:**
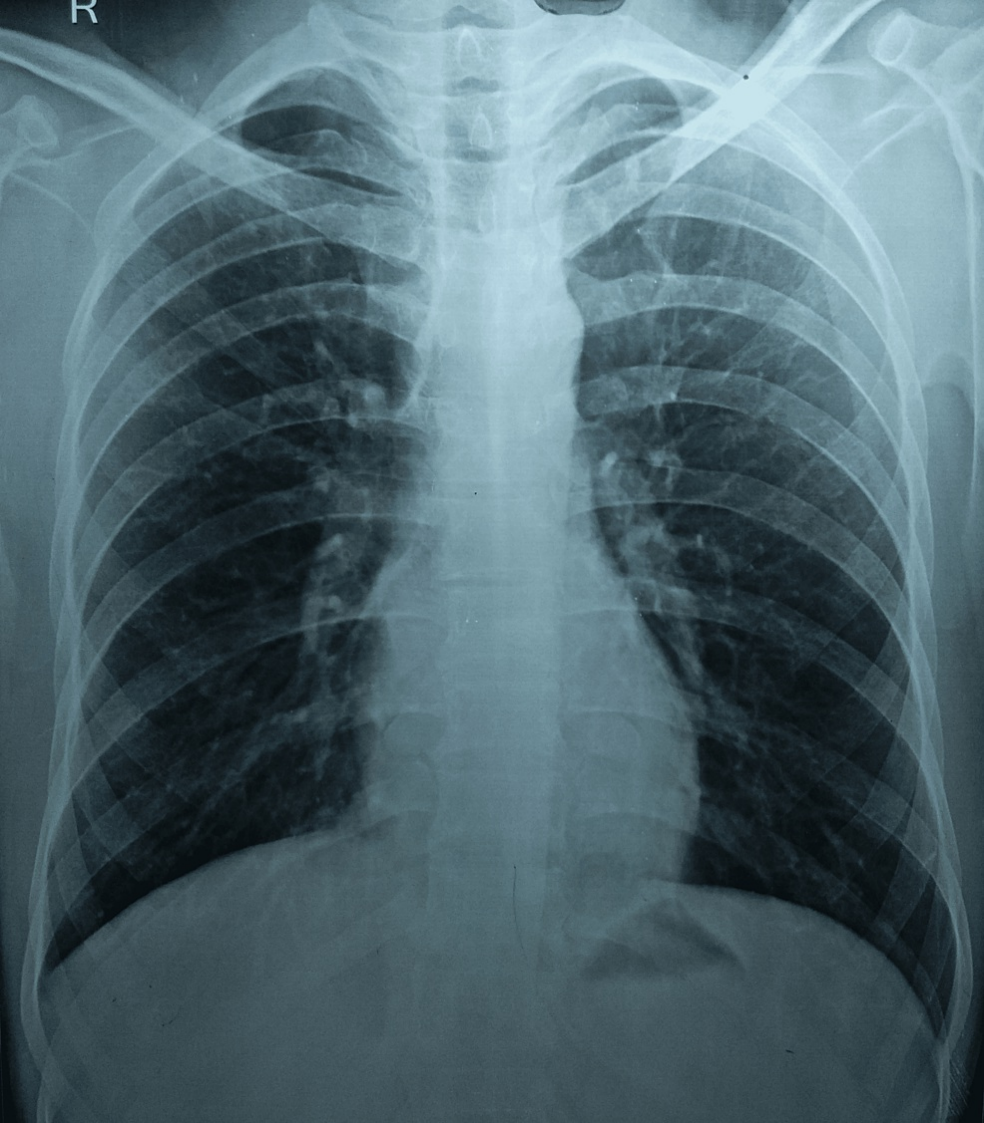
A normal chest radiograph (P-A view) P-A, Postero-anterior

A detailed laboratory examination revealed hemoglobin of 11.9 g/dl and a raised erythrocyte sedimentation rate of 61 mm in the first hour. All other laboratory parameters were within normal limits. The hepatitis panel (hepatitis A, B, and C) was negative, and HIV status was negative for both HIV I and II. The tuberculin skin test was positive, with an induration of 21 mm. The sputum smear microscopy and cartridge-based nucleic acid amplification test of the induced sputum were negative. The patient underwent fine-needle aspiration cytology of the anterior cervical lymph node. The results were suggestive of a few granulomas consisting of epitheloid cells, lymphocytes, and occasional Langhans giant cells in a background of scanty necrosis. Ziehl-Neelsen staining was positive for acid-fast bacilli. Therefore, a diagnosis of drug-sensitive right cervical lymphadenitis was made, and he was initiated on first-line anti-tubercular chemotherapy. As a part of the universal drug susceptibility guidelines of the national program and clinical suspicion of drug resistance in this case, an excisional biopsy was performed, and samples were sent for a cartridge-based nucleic acid amplification test, a line-probe assay, and culture. The results were surprising, with the detection of *Mycobacterium tuberculosis* on a cartridge-based nucleic acid amplification test (no resistance to rifampicin) and a line-probe assay (sensitive to rifampicin but high-level resistance to isoniazid on the katG gene). The culture (liquid culture system, Bactec Automated Blood Culture System (Becton, Dickinson and Company, Franklin Lakes, New Jersey, United States) grew *Mycobacterium tuberculosis* with resistance to isoniazid and no resistance to any other first-line anti-tubercular drug. An extended second-line drug resistance test was not suggestive of any other resistance. A chest radiograph was normal (Figure 2).

A final diagnosis of primary isoniazid mono-resistant extrapulmonary tuberculosis of the multiple right cervical lymph nodes was made, and a pre-treatment evaluation of the patient was done, per the national guidelines. As the pre-treatment evaluation was within normal limits, he was initiated on the isoniazid-mono regimen per the national guidelines (Table 1).

**Table 1:** Isoniazid-mono regimen per the national guidelines

Drug	Route of administration	Dose
Rifampicin	Per oral	600 mg
Ethambutol	Per oral	1200 mg
Pyrazinamide	Per oral	1750 mg
Levofloxacin	Per oral	1000 mg

He was counseled for a high-protein diet and hygienic lifestyle. His treatment was initiated for six months, but being a daily-wage worker he requested to be transferred to a different state, and therefore his last follow-up was not done. Although the national electronic data portal (Nikshay) had his treatment outcome as treatment complete.

## Discussion

Tuberculosis is a constant challenge to public health [1]. The disease is an outcome of the inhalation of infected aerosols [7]. Mostly, it is reported as drug-sensitive tuberculosis, which is managed with first-line anti-tubercular drugs like isoniazid, rifampicin, pyrazinamide, and ethambutol [8]. However, drug-resistant tuberculosis can manifest as isoniazid-resistant tuberculosis, rifampicin mono-resistant tuberculosis and multidrug-resistant tuberculosis, pre-extensively drug-resistant tuberculosis that is resistant to rifampicin and any fluoroquinolone, and extensively drug-resistant tuberculosis that is resistant to rifampicin plus any fluoroquinolone, plus a minimum of one of the drugs, i.e., bedaquiline and linezolid [8].

Isoniazid is a cornerstone of anti-tubercular chemotherapy, mainly due to its prompt bactericidal activity, low cost, and fewer side effects compared to other anti-tubercular drugs [1,9]. Besides, drug resistance is a man-made issue and is mostly a result of improper administration of anti-tubercular drugs [10]. Therefore, it is essential that a detailed history of prior exposure to anti-tubercular drugs be obtained before the start of treatment.

Globally, tuberculosis resistant to isoniazid and susceptible to rifampicin is the most prevalent drug-resistant form (besides streptomycin resistance), with estimates going to 7% among newly diagnosed tuberculosis cases and 8-11% among previously treated tuberculosis cases [11]. Primary isoniazid resistance is rare, but reports of this type of drug resistance are available in the literature [12]. The global prevalence of isoniazid mono-resistance is between 0.0 and 9.5% [13]. Furthermore, the same in extrapulmonary cases with no prior history or concomitant pulmonary involvement is also not available.

The diagnosis of primary isoniazid-resistant tuberculosis is difficult. Often, the clinical presentations overlap with drug-sensitive forms. As seen in this case, there were no constitutional symptoms of tuberculosis, which led to an initial diagnosis of drug-sensitive right cervical lymphadenitis. However, the well-defined guidelines of the national program, which mandate universal drug susceptibility testing of all the diagnosed cases, helped in establishing the correct diagnosis [8]. This also helped in reducing the time from the initial presentation to the establishment of a final diagnosis and the initiation of management. Nevertheless, a missed or delayed diagnosis could result in unfavorable outcomes and fatalities, as isoniazid mono-resistance could progress to severe forms of multidrug-resistant tuberculosis [11].

In a study on 7578 isoniazid mono-resistant tuberculosis cases from 24 countries, Karo et al. reported that there was a lower likelihood of successful treatment in cases of tuberculosis when isoniazid resistance was present [14]. In a 15-year-long study from Mexico, Báez-Saldaña et al. reported that there was a higher chance of treatment failure and fatalities in cases with isoniazid mono-resistance [13].

Primary isoniazid-resistant tuberculosis should always be kept in mind when a case similar to the present is reported. It is essential that drug susceptibility testing be done in all diagnosed and highly suspected cases. Finally, this was a very rare case, as no similar case is available where primary extrapulmonary isoniazid mono-resistance is reported in a cervical lymphadenitis patient. The scarcity of data on extrapulmonary isoniazid mono-resistance would encourage the reporting of similar cases from high-burden settings. This will probably help in changing or making new management algorithms.

## Conclusions

A case of primary extrapulmonary isoniazid mono-resistance is reported in a cervical lymphadenitis patient with no history of tuberculosis or pulmonary involvement. A very high degree of suspicion is essential to diagnose such cases. Primary care physicians should be aware of these conditions, especially in endemic countries, as this will not only reduce the chances of treatment failure but also prevent life-threatening consequences.
